# Use of Early-Onset Sepsis Risk Calculator for Neonates ≥ 34 Weeks in a Large Tertiary Neonatal Centre, Saudi Arabia

**DOI:** 10.7759/cureus.14620

**Published:** 2021-04-21

**Authors:** Roya Huseynova, Latifa Bin Mahmoud, Fahad Hamad Aljobair, Ogtay Huseynov, Halima Career, Parameaswari P Jaganathan, Adli Abdelrahim, Faisal A Abduljabar Alaklobi

**Affiliations:** 1 Neonatal Intensive Care Unit, King Saud Medical City, Riyadh, Riyadh, SAU; 2 Infectious Diseases Department, King Saud Medical City, Riyadh, Riyadh, SAU; 3 Neurosurgery Resident, Azerbaijan Medical University, Baku, AZE; 4 Obstetrics and Gynecology, King Saud Medical City, Riyadh, Riyadh, SAU; 5 Research and Innovation Center, King Saud Medical City, Riyadh, Riyadh, SAU

**Keywords:** early-onset sepsis, kpc calculator, infection risk calculator, eos risk calculator, infant, late preterm, antibiotic, neonatal sepsis calculator

## Abstract

Early-onset sepsis (EOS) refers to sepsis with onset before 72 hours of life. Kaiser Permanente Calculator (KPC) or EOS risk calculator is an advanced multivariate risk model for predicting EOS in infants.

Objective

To examine the EOS risk calculator effect for predicting neonatal EOS, the necessity for laboratory tests, antibiotic usage, and length of hospital stay among the term and late-preterm newborns.

Method

In this cross-sectional study, we evaluated 44 cases of neonates ≥34 weeks of gestation started on empiric antibiotics within 72 hours after birth due to suspected EOS at the neonatal intensive care unit (NICU). The study site is a 1,500-bed teaching hospital, with around 4,500 annual deliveries, 70 beds in the level II and level III tertiary care NICU. We calculated the risk of the incidence of EOS as one per 1000 live births. Then we retrospectively calculated the probability of neonatal early-onset infection at birth based on the EOS risk calculator and assigned each neonate to one of the recommended categories of the calculator. The primary outcome was to evaluate the infection risk calculator's effect for predicting neonatal EOS and antibiotic usage among the term and late-preterm newborns ≥34 weeks of gestation.

Results

In our data, EOS calculator showed unnecessary antibiotic usage for 12 (27.3%) neonates [relative risk reduction (RRR) 27.2%; 95% confidence interval (CI) 20.3% - 35.7%)]. EOS risk calculator implementation may decrease in the number of NICU admission (RRR 20.4%; 95% CI 14.3% - 28%), laboratory tests (RRR 20.4%; 95% CI 14.3% - 28%), and length of stay (RRR 25%; 95% CI 38% - 95%).

Conclusion

EOS calculator could be considered a strategic and objective implementation for managing EOS that can limit unnecessary laboratory tests, reduce antibiotic usage, and length of stay related to EOS. Our findings ensure a multicenter, randomized study evaluating the safety and general use of the calculator for EOS sepsis in Saudi Arabia's clinical practice.

## Introduction

Early-onset sepsis (EOS) remains a severe problem for neonates associated with the significant cause of infant morbidity and mortality in both high and low-income countries [1]. According to World Health Organization (WHO) in 2015, the incidence of EOS overall worldwide was one to 5/1000 live births, with the mortality rate of late preterm babies ≥ 35 weeks around 2-3% [2]. Early-onset neonatal sepsis incidence in Arab countries is 0.5-1.4 per 1000 live births [3].

The EOS risk factors include chorioamnionitis, maternal group B streptococcus (GBS) colonization, inadequate intrapartum antibiotic prophylaxis for GBS, and prolonged rupture of membranes. About 60% of term babies with EOS need admission for respiratory and cardiovascular support, although EOS's clinical manifestation may appear later. 

Clinical presentations of sepsis may include acidosis, tachycardia or bradycardia, hypoglycemia, jaundice, feeding intolerance, systemic hypotension, lethargy or irritability, respiratory distress, and apnea. However, these nonspecific findings can also be related to non-infectious factors that cause physicians to determine who should receive antibiotics and lead to the overuse of empiric antibiotics among infants even with widely applied antibiotic stewardship programs [4]. 

Additionally, admission to the neonatal intensive care unit (NICU) may interrupt breastfeeding and parental bonding. Mukhopadhyay et al. demonstrated that EOS evaluation in asymptomatic infants results in delayed breastfeeding initiation almost four-fold and increased formula supplementation two-fold [5].

Finally, around 40% of all neonates were exposed to antibiotics before the delivery due to maternal surgical prophylaxis in cesarean deliveries, maternal GBS intrapartum antibiotic prophylaxis (IAP) proved, and suspected chorioamnionitis [6]. Therefore, neonatal health providers should consider the risk and benefit of initiating antibiotic therapy in newborns with suspected EOS and the duration of antibiotics course in the absence of culture-proved infection. For that, a combination of evidence-based antibiotics programs and clinical approaches can be beneficial in reducing antibiotics use [4]. 

To decrease unnecessary hospital admission and antibacterial treatment to well-appearing infants, researchers at Kaiser designed the EOS risk calculator, a robust logistic regression model, Kaiser Permanente Calculator (KPC) that provides individualized evaluations of early-onset sepsis risk in neonates ≥ 34 week's gestation [7]. The EOS risk calculator provides an early-onset sepsis risk estimate for each neonate based on the five objective maternal risk factors and four clinical neonatal risk factors. It categorized neonates into three levels of risk with a correlated recommendation, like laboratory tests, start or not to start antibiotic treatment. The EOS calculator is a freely available online validated tool; however, it lacks standard guidelines for its use, which provides some discomfort with the practice change [8]. 

Based on the available data, the EOS risk calculator's implementation can significantly decrease the unnecessary use of antibiotics in asymptomatic neonates in the first 72 hours [9]. Furthermore, decreased administration of antibiotics with the EOS risk calculator may reduce the rate of hospital admission and costs. The usage of neonatal EOS calculator is increasing in various countries and continents, including Australia, the USA, and Europe.

Although this tool may decrease antibiotic administration to neonates at risk for EOS sepsis, related side effects, and shorten the duration of hospital stay, it has not yet been validated in Saudi Arabia. The calculator can be served as the tool of change away from the previously recommended practice, may decrease the need for diagnostic investigations and empirical therapy in neonates in Saudi Arabia.

Evidence supporting the effectiveness and safety of the calculator is an essential issue before considering its implementation. Although the available evidence regarding the neonatal calculator's safety is not sufficient, it did not show its inferiority compared to used conventional treatment policies [9]. 

Another critical point is that the EOS risk calculator should be considered only as a supportive tool that providers can use after looking at the overall clinical picture to decide about the EOS evaluation and further management.

Given the significant safe reduction in antibiotic usage and investigations for infection, we decided to investigate the possibility of applying the EOS risk calculator tool in our center. Therefore, our objective was to evaluate whether implementing the EOS risk calculator in neonates with suspected early-onset sepsis would decrease antibiotic administration within 24-72 hours compared to the institutional policy.

## Materials and methods

From February 1, 2020, through June 30, 2020, there were 2063 neonates born at or after 34 weeks of gestation in a single tertiary teaching center. From those, we identified 44 cases started on empiric antibiotics within 72 hours after birth due to suspected EOS and placed them on the web-based neonatal EOS risk calculator [7]. We observed that the EOS calculator identified that 27.3% of the neonates should not be prescribed antibiotics, with a 10% margin of error, 90% power, 95% confidence level & 5% type I error; the minimum required sample size is 29.

**Figure 1:**
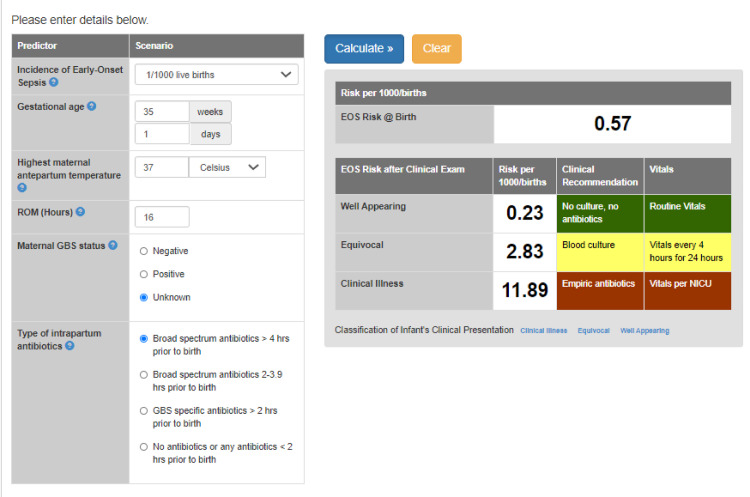
EOS risk calculator (example of the calculation of the risk of the neonatal EOS)

Information required for calculating EOS scores included gestational age (GA), highest maternal antepartum temperature, rupture of membranes, maternal GBS status, onset, and type of intrapartum antibiotics. The example of the use of the neonatal EOS risk calculator is demonstrated in Figure 1.

Neonatal clinical parameters used for assessing the risk for early-onset sepsis by EOS calculator include Apgar scores, oxygen saturation, heart rate, respiratory rate, temperature, signs of respiratory distress, mode of respiratory support, inotropic drugs, and presence of the hypoxic-ischemic encephalopathy (HIE). According to these clinical presentations, we categorized neonates into one of the infection risk calculator's recommended states: well-appearing, equivocal, or clinical illness groups [7]. The EOS risk score then incorporated the clinical finding of each case to determine the appropriate management plan. We used the incidence of EOS as one per 1000 live births, which was considered the likely risk at our institution based on the calculated prevalence rate of EOS for the previous year.

Additional neonatal data from medical reports included gender, birth weight, antibiotic usage, length of hospital stay, and mortality. The infants with suspected EOS were managed according to the unit guidelines based on the centers for disease control (CDC) 2010 guidelines [10].

The unit guidelines recommend management for neonates with suspected EOS with intravenous ampicillin and gentamycin up to 48 hours if culture is negative, otherwise to continue according to the patient's clinical condition. For those neonates with hypoxic-ischemic encephalopathy, acute renal injury, and concern for infection, unit guidelines suggest the possible substitution of gentamycin with cefotaxime. Diagnosis of suspected or diagnosed chorioamnionitis was considered if it was documented in the maternal medical file. Prolonged rupture of membranes was considered if it lasted longer than 18 hours between the time of rupture and the delivery time.

Neonates born less than 34 weeks gestation and neonates born at term not received antibiotics were excluded. Maternal microbiologic data, antibiotic exposure, intrapartum temperature, GBS status, duration rupture of the membrane, we obtained from maternal medical records. 

Early-onset sepsis is defined as blood or cerebrospinal fluid (CSF) culture-confirmed infection with a pathogenic bacterial species with onset before 72 hours of age [11]. The organisms most frequently involved in early-onset neonatal sepsis (EOS) are group B streptococcus (GBS), Escherichia coli, Listeria monocytogenes, Coagulase-negative Staphylococcus, and Haemophilus influenza.

Empirical antibiotic exposure for EOS was defined as antibiotics treatment initiated before culture reports were known and within 72 hours of age. The recommendations for antibiotic therapy were retrospectively compared to both methods: infection risk calculator and unit guidelines for suspected EOS, which did not affect the clinical management. 

The primary outcome was to determine the proportion of neonates who do not need antibiotic administration within 24-72 hours by using the EOS risk calculator. The secondary outcome was to assess the advantage of implementing a neonatal EOS risk calculator in decreasing blood investigations, duration of antibiotics treatment, and length of hospital stay.

The data was analyzed using SPSS 25.0 (IBM SPSS Statistics for Windows, Version 25.0. Armonk, NY: IBM Corp). We classified the 44 neonates into three categories as per clinical assessment. Continuous variables presented as Mean ± standard deviation (SD). The Z-test for proportions used for the nominal variables, the non-parametric independent sample Kruskal-Wallis test was used for the deviated variables and the analysis of variance (ANOVA) for comparing the means of three groups. Parametric Pearson coefficient of correlation was estimated between clinical assessment and EOS calculator. Bland-Altman plot of agreement and linear regression model were predicted. All the above tests were applied and observed for their statistical significance at a 5% level.

## Results

The study included 44 infants born at 34 weeks gestation or later. Of these 44 neonates, 20.4% did not require admission to the NICU based on their calculated clinical risk assessment using the online neonatal EOS calculator [7]. The median duration of antibacterial therapy was six days. We identified no proven positive result of blood culture.

From 44, 23 (52.2%) infants were born through cesarean section. We detected positive group B streptococcus (GBS) status in three (6.8%) mothers, rupture of membrane ≥ 18 hours in 10 (22.7%), maternal antibacterial therapy in 19 (43.2%), and suspected maternal chorioamnionitis present in two (4.5%) of 44 cases.

Maternal characteristics (Table 1) revealed that the rate of cesarean section delivery was 17 out of 23 (73.9%), premature rupture of membrane ≥ 18 hours was seven out of 10 (70.0%) and maternal antibiotics administered among 13 out of 19 (68.4%) were higher compared to the equivocal and well-appearing groups.

**Table 1:** Maternal variables for clinically assessed neonates

Maternal Variables	Clinically ill appearing group of neonates	Equivocal appearing group of neonates	Well appearing group of neonates
	n=29 (66.0)	n=9 (20.4)	n=6 (13.6)
Mode of Delivery, n (%):			
Cesarean	17 (58.6)	3 (33.3)	3 (50.0)
Normal Vaginal Delivery	12 (41.4)	6 (66.7)	3 (50.0)
Rupture of Membrane (hours), n (%):			
< 18	22 (75.8)	7 (77.7)	5 (83.3)
≥ 18	7 (24.2)	2 (22.2)	1 (16.6)
Diabetes, n (%)	4 (13.8)		1 (16.6)
Group B Streptococcus positive mothers, n (%)			3 (50.0)
Maternal antibiotic use, n (%):	13 (44.8)	2 (22.2)	4 (66.6)
Ampicillin	5 (17.2)	1 (11.1)	2 (33.3)
Cefazolin	7 (24.1)	1 (11.1)	1 (16.6)
Erythromycin & Cefazolin	1 (3.4)		1 (16.6)
Chorioamnionitis, n (%)	1 (3.4)		1 (16.6)

Suspected chorioamnionitis was present in two (4.5%) of 44 cases; one case was observed in clinically well-appearing and another in the clinically ill neonate's groups. All GBS-positive mothers (100.0%) were in the well-appearing infant group. 

Neonate variables presented in Table 2 showed that among the 44 infants, 36 (81.8%) were appropriate gestational age. All patients received antibiotic therapy within 12 hours of age. The antibiotics duration exceeded three days was seen in seven (16.0%) cases; six 86.0%) of them were in the clinically ill group. 

**Table 2:** Neonate variables in clinically ill, equivocal, and well-appearing groups

Neonate variables	Clinically ill appearing group of neonates	Equivocal appearing group of neonates	Well appearing group of neonates
	n=29 (66.0%)	n=9 (20.4%)	n=6 (13.6%)
Gender, n (%): Male	19 (66.5)	4 (44.4)	2 (33.3)
Female	10 (33.5)	5 (55.6)	4 (66.6)
Gestational Age (GA) (weeks), mean (SD)	37.2 (0.5)	36.2 (1.03)	37.6 (0.33)
Small for GA, n (%)	5 (17.2)	2 (22.2)	
Appropriate for GA, n (%)	23 (79.3)	7 (77.8)	6 (100.0)
Large for GA, n (%)	1 (3.4)		
Birth weight (kg), mean (SD)	2.8 (0.17)	2.1(0.31)	2.4 (0.12)
Apgar scores mean (SD):			
at 1^st^ minute	4.9 (0.75)	6.2 (0.85)	7.6 (0.30)
5^th ^minute	7.4 (0.35)	8.2 (0.47)	8.9 (0.10)
10^th^ minute	7.8 (0.24)	8.2 (0.47)	9.0 (0.04)
Respiratory rate at birth, mean (SD)	63.2 (2.98)	53.7 (1.60)	40.1 (1.06)
Heart rate at birth, mean (SD)	158.3 (3.64)	151.2 (2.49)	140.6 (5.57)
Oxygen saturation in first 2 hours of age (%), mean (SD)	91.9 (0.77)	93.7 (0.62)	95.6 (0.27)
Temperature (C°), mean (SD)	36.9 (0.03)	36.9 (0.10)	37.0 (0.10)
EOS risk by EOS calculator after clinical exam			
Mean (SD)	20.4 (9.10)	2.25 (0.87)	0.13 (0.03)
EOS risk by EOS calculator			
Mean (SD)	1.25 (0.58)	0.44 (0.17)	0.23 (0.09)
Blood culture: no growth, n (%)	29 (100.0)	9 (100.0)	6 (100.0)
C-reactive protein (CRP)	26 (89.6)	5 (55.5)	4 (66.6)
Antibiotics, n (%):	29 (100.0)	9 (100.0)	6 (100.0)
Ampicillin, Gentamycin	26 (89.6)	9 (100.0)	6 (100.0)
Ampicillin, Cefotaxime	3 (10.3)		
Antibiotic therapy started less than 12 hours of age, n (%)	29 (100.0)	9 (100.0)	6 (100.0)
Duration of Antibiotics (days), n (%):			
≤ 3	23 (79.3)	8 (88.9)	6 (100.0)
>3	6 (20.7)	1 (11.1)	
Room Air, n (%)			6 (100.0)
Respiratory support in first 2 hours of age:			
Nasal Cannula (2 L/min)	10 (34.5)	9 (100.0)	
Continuous Positive Airway Pressure (CPAP)	5 (17.2)		
Mechanical ventilation (MV)	12 (41.3)		
High Flow Nasal Cannula (HFNC)	2 (6.9)		
Seizures	6(20.7)		
Length of stay (days), n (%):			
≤ 3	4 (13.8)	2 (22.2)	5 (83.3)
>3	25 (86.2)	7 (77.8)	1 (16.6)
Mortality, n (%)	2(6.9)		
Causes for admission, n (%):			
GBS positive mothers			3 (50.0)
Transient Tachypnea of Newborn (TTN)	9(31.0)	5(55.6)	1 (16.6)
Perinatal Depression	1 (3.4)	2 (22.2)	1 (16.6)
Respiratory distress syndrome (RDS)	6 (20.7)	2 (22.2)	
Congenital pneumonia	2 (6.9)		
Hypoxic Ischemic Encephalopathy (HIE)	6 (20.7)		
Meconium Aspiration Syndrome (MAS)	4 (13.8)		
Suspected chorioamnionitis	1 (3.4)		1 (16.6)

The clinically well-appearing group includes 6 (16.7%) neonates with a mean ± SD Apgar scores of 7.8 ± 0.75, 8.6 ± 0.35 on 1st and 5th minutes, respectively. The vital signs and clinical appearance of the neonates did not reveal any significant abnormal findings.

None of these patients required any respiratory support, and average oxygen saturation of 95.6% in the first two hours of life was reported while on room air. The main cause of admission was maternal antibiotic use four (66.6 %) and three (50.0%) due to maternal GBS colonization. All pregnant women in these groups received an antibacterial treatment course more than two hours before the deliveries. Blood culture requested in six (100,0%) newborns. 

All infants in the well-appearing group received antibiotic therapy and laboratory investigations; however, the EOS calculator didn't recommend antibacterial treatment. One-half of well-appearing infants admitted and received antibiotic therapy due to positive maternal GBS status. The mean±SD calculated risk sepsis by EOS calculator for these cases was 0.23 ± 0.09 per 1000 live births without adjustment the clinical data. 

The equivocal group includes nine (20.45 %) of the total study cases. The patients in this group tend to have lower mean gestational age 36.2 ± 1.3 weeks compared to clinically ill and well-appearing groups 37.2 ± 0.5 and 37.6 ± 0.3 weeks respectively, lower Apgar scores and oxygen saturation in the first two hours of life 93.7% ± 0.6% compared to neonates of the well-appearing group. The leading cause of admission was transient tachypnea of the newborn (TTN) (55.6%). All patients received support with a nasal cannula (2 L/min) that was ceased within 24 hours and antibiotic therapy for more than three days. In the equivocal group, all patients received antibiotic treatment (100.0%) and laboratory tests (100.0%), while the EOS calculator suggests treatment only for three (33.3%) and laboratory investigations for 66.7%. The remaining 33.3% calculator didn't recommend either empirical antibiotics or laboratory investigations but just qualified observations every four hours. The mean ± SD calculated risk sepsis for these cases was 0.44 ± 0.17 per 1000 live births without adjustment to clinical status. 

Clinically-ill group infants account for 66.0% of cases, 79.3% of those were term infants with an appropriate for gestational age weight. No apparent significant maternal risk factors were detected in this group, except for one case of chorioamnionitis (3.4%). All mothers had unknown GBS status. The patients in this group tended to have significantly lower Apgar scores with a mean of 4.9±0.75 on the first minute, 7.4 ± 0.35 at the fifth minute and 7.8±0.24 at the 10th minute, more tachypneic (P=0.000), tachycardic (P=0.000) and lower oxygen saturation in 91.9% (P=0.003) in the first two hours of life compared to the other groups. All cases presented with respiratory distress and 43.4% of them required mechanical intubation. 

Reasons for admission included mainly transient TTN 31.0%, respiratory distress syndrome (RDS)-20.7%, hypoxic-ischemic encephalopathy (HIE)-20.7%, meconium aspiration syndrome-13.8%, and congenital pneumonia-6.9%. The mortality was 6.9% that was associated with causes other than EOS. Twenty-nine cases received antibiotics and passed through the EOS-related laboratory tests. EOS calculator recommended empiric antibiotics in all 29 patients.

The non-parametric independent sample Kruskal-Wallis test (P=0.345) performed on the distribution of EOS calculator across categories of clinical assessment was not significant and when performed on the distribution of antibiotics given to baby (P=0.000) and distribution of length of stay (P=0.001) across categories of clinical assessment was found to be statistically different. 

**Figure 2:**
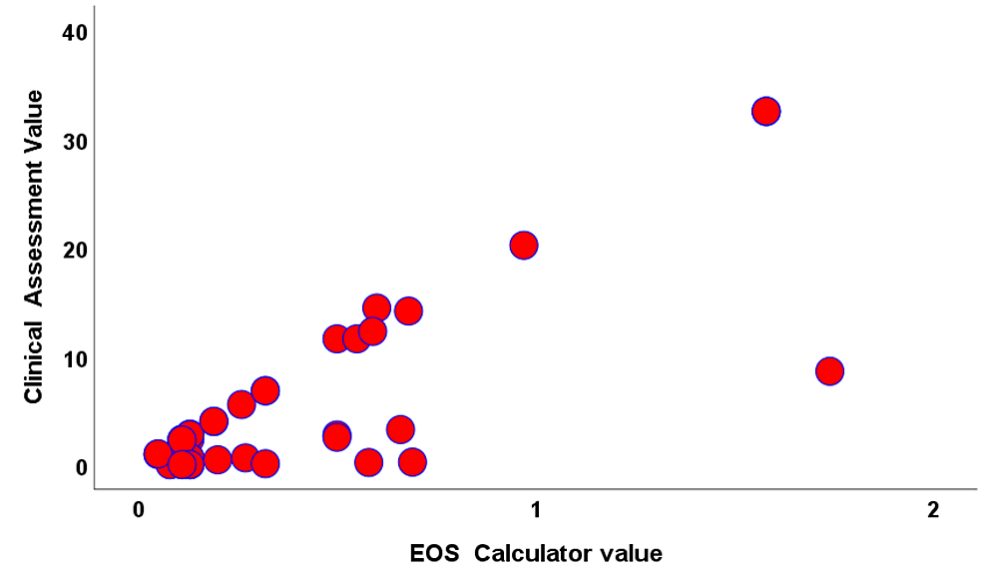
Scatter Diagram of the two assessment values

The magnitude of the correlation between the EOS risk calculator value and the clinical assessment value was estimated by Pearson coefficient r =+0.971 (P=0.000), and Figure 2 displays the scatter diagram for the positive relationship between two EOS risk assessment values.

**Figure 3:**
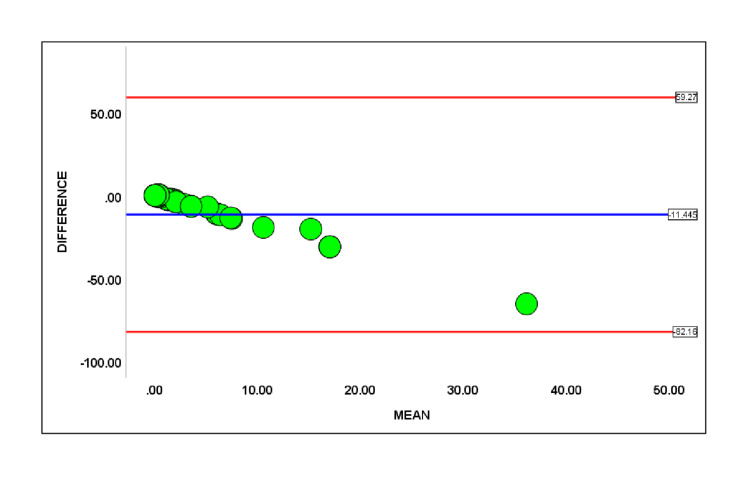
Bland-Altman Plot for the difference between clinical local practice and applied EOS risk calculator

Bland-Altman plot (Figure 3) measures the agreement between two EOS risk assessment values displayed in the x-axis against their difference with the mean reference line (-11.445) for the y-axis in the blue line and the limit of agreement 95% confidence interval (-82.16 to 59.27) in the red line. 

Further, these two variables' mean' and 'difference' used in the linear regression model, which led to a regression coefficient β = -1.767(P=0.000), implying a proportional bias exists between the two measures within the limit of agreement. We found a statistically significant reduction of antibiotic use and laboratory investigations in the equivocal and well-appearing groups after implementing the EOS risk calculator (Table 3). The sepsis calculator did not recommend empiric antibiotics for 27.3% (P=0.0003) out of 44 studied cases.

**Table 3:** Antibiotic therapy and laboratory tests in neonates (n=44) recommended by the EOS risk calculator

Parameters	Antibiotic use recommended by the EOS risk calculator			Laboratory tests recommended by the EOS risk calculator		
	Equivocal appearing group of neonates	Well appearing appearing group of neonates	Total	Equivocal appearing group of neonates	Well appearing group of neonates	Total
Before use of the EOS risk calculator	9	6	44	9	6	44
After use of The EOS risk calculator	3	0	32	6	0	35
Difference in proportion , n (%)	66.7	100.0	27.3	33.3	100	20.5
P-value	0.0035*	0.0009*	0.0003*	0.0654	0.0009*	0.0017*
95% Confidence Interval	23.4 - 87.9	44.7 - 100	12.5 - 44.5	3.4 - 64.5	44.7 - 100	7.5 - 36.4

## Discussion

Antibiotic overuse and resistance to commonly used antibiotics is a global problem that overhangs novel medicine achievements. One of the most common admission diagnoses utilized by neonatal health care providers is "rule out sepsis," despite the low incidence of proven culture-positive sepsis.

Implementing the EOS calculator for predicting neonatal sepsis and close clinical observation might decrease NICU admissions in healthy-appearing infants [12,13]. Furthermore, a significant reduction in antibiotic therapy usage from 5% to 2.6% achieved with the EOS calculator usage in several studies [9,14,15]. Also, calculator usage has been associated with a significant reduction of health utilization and associated costs (15).

 The presented study is the first report from the Kingdom of Saudi Arabia on implementing the EOS calculator among newborns.  Our study's results were comparable with other studies evaluating the infection calculator's validity [16,17]. By applying the EOS calculator in well-appearing and equivocal groups, we found that antibiotic usage could significantly decrease from 100.0% to 0 (P=0.0009) and from 100.0% to 33.3% (P=0.0035), respectively. Neonates admitted for EOS evaluation did not have culture-confirmed sepsis.

Another critical point is determining the adequate antibiotics duration course without any positive cultures. Simonsen et al. reported that around 10% of all neonates are investigated for presumed EOS; however, only approximately 5% have positive cultures [18]. However, in highly suspected clinical cases with negative culture, antimicrobial therapy may continue for seven to 10 days [19]. 

We found that the average length of stay in our study among the neonates in the well-appearing group was three days, in equivocal eight days, and the ill-appearing group twelve days. However, clinical conditions of all cases in well-appearing and equivocal groups improved within the first 24 hours; all of them maintained an appropriate oxygen saturation while on room air after two hours of age. 

We also found that the main possible reason for an extended NICU stay among neonates (well-appearing and equivocal groups) was requested blood culture and c-reactive protein (CRP), which resulted in the further extension of the antibiotic exposure for an average of 72 hours in both groups. 

Elimination of the routine laboratory tests like complete blood count (CBC) and CRP are supported by their low sensitivity in predicting EOS in late-preterm and term newborns in several studies [19,20]. Furthermore, every prick for blood collection and peripheral catheters' insertion for antibiotics administration is painful. It is well known that repeated painful exposures can potentially adverse events like physiologic instability, altered stress response system, and brain development [21,22].

Dhudasia et al. reported a reduction of laboratory tests almost by 50% in infants admitted to the NICU and around 80% among the well-appearing infants with the utilization of the multivariable risk prediction models of EOS [23]. When we applied the EOS calculator, we found that laboratory evaluations for studied cases can be decreased from 100.0% to 66.7% (P=0.0035) in equivocal; from 100.0% to 0.0% (P=0.0009) in well-appearing groups with a total reduction of 20.5% (P=0.0017) in these groups. 

Carola et al. reported that some cases with culture-proven EOS could be missed with an infection calculator [24]. At this point, the primary consideration was given to" missed cases" that were defined as cases in which the infection calculator did not recommend antibiotics, but the National Institute for Health and Care Excellence (NICE) guidelines did [14]. However, calculator editors identified that EOS cases due to extended ongoing evaluations of the neonates might not have been truly missed due to the neonates' comprehensive continuous assessment [25]. Guidelines by the center for disease control and prevention (CDC) recommend laboratory tests (blood culture and CBC) and empiric antibiotic therapy for 48 hours of newborns born to mothers with chorioamnionitis (suspected or confirmed); however, these recommendations are being re-evaluated nowadays [26]. 

What about antibiotic treatment in healthy-appearing infants born to mothers with chorioamnionitis? We determined one case born to a mother with chorioamnionitis in the ill- and another in a well-appearing category. The management of newborn infants at risk for EOS continues to be controversial, especially when clinically well. 

Based on our hospital protocol, all newborns born to mothers with suspected or proven chorioamnionitis were admitted to the NICU for investigations and received suspected EOS treatment for at least 48 hours, regardless of clinical presentation. We found that the leading cause of admission to NICU in our study was primarily non-infectious, and treatment was started due to "rule out sepsis."

Based on the presented data, we considered that neonatal practice focused on the empiric treatment of late preterm and term neonates at risk for EOS cannot prevent EOS and should not be used. Currently, the only proven preventive EOS approach is the appropriate maternal intrapartum antibacterial treatment [27].

However, from another side, the safety of that tool's use in neonates can be a significant concern due to the risk of possible missing cases of EOS and potential delays in antibiotic therapy administration. Kuzniewicz et al. concluded that a substantial reduction in antibiotic usage is significantly overweighed compared to a possibility of delay in antibiotic therapy [9]. Furthermore, no significant morbidity or mortality related to culture-proven EOS or readmission or delay in antibiotic treatment was reported using the infection calculator [28].

Our experience showed that the calculator's probability of decreasing antibiotics use is impressive and may reduce antibacterial treatment from 100.0% to 72.8% (P=0.0003).

In Saudi Arabia, significant over-treatment with antibiotics for suspected neonatal EOS represents the insistent clinical problem, causing risks for infants as it leads to more nosocomial infections and antibiotics resistance [29]. Implementation of an EOS risk calculator would potentially improve clinical practice and limit the unnecessary usage of antimicrobials in Saudi Arabia.

This study has several limitations. It was a short duration, retrospective study conducted in a single center. The sample size was small, which may have been insufficient to detect culture-positive sepsis due to the disease's low incidence rate. Furthermore, a larger prospective trial is needed to evaluate the neonatal EOS calculator on the incidence of mortality and readmission rate. We could not follow proper serial physical examination after birth because the infection calculator was applied in a theoretical manner that may differ in real-time clinical scenarios.

## Conclusions

Implementation of neonatal sepsis calculator is associated with a reduction in laboratory tests, antibiotic use, and length of stay related to EOS evaluation. Our data showed that this simple clinical decision support tool could be considered a strategic and objective implementation of managing EOS that can reduce antibiotic usage by more than 27%. These findings ensure a multicenter, randomized study evaluating the safety and general use of the EOS risk calculator in clinical practice in Saudi Arabia. 
